# Acquired resistance to anti-PD1 therapy: checkmate to checkpoint blockade?

**DOI:** 10.1186/s13073-016-0365-1

**Published:** 2016-10-25

**Authors:** Jake S. O’Donnell, Mark J. Smyth, Michele W. L. Teng

**Affiliations:** 1 Immunology in Cancer and Infection Laboratory, QIMR Berghofer Medical Research Institute, Herston, 4006 QLD Australia; 2 Cancer Immunoregulation and Immunotherapy Laboratory, QIMR Berghofer Medical Research Institute, Herston, 4006 QLD Australia; 3 School of Medicine, The University of Queensland, Herston, 4006 QLD Australia

## Abstract

Anti-programmed cell death 1 (PD1) immunotherapies are among the most effective anti-cancer immunotherapies available; however, a large number of patients present with or develop resistance to them. Unfortunately, very little is known regarding the mechanisms of resistance to such therapies. A recent study sought to identify mutations associated with resistance to anti-PD1 therapy. Results from this study demonstrated that mutations which affected the sensitivity of tumor cells to T-cell-derived interferons, and mutations limiting tumor-cell antigen presentation, could cause acquired resistance. These findings have significant implications for understanding the mechanisms by which anti-PD1 therapies exert their efficacy, comprehending why and how some patients acquire resistance over time, and ultimately guiding the development of combination therapies designed to overcome, or potentially prevent, the development of acquired immunotherapeutic resistance.

## Cancer immunotherapy and immune checkpoints

Advances in cancer immunotherapy have resulted in remarkable success in the treatment of a variety of human cancers. Conceptual developments, such as the understanding that immune responses are routinely generated against tumor-specific neoantigens (derived from proteins mutated in the cancer) and that these responses are usually limited by immunosuppressive tumor microenvironments, have been key to the development of immunotherapies capable of promoting immunological control of tumor progression. Such therapies can act either passively, by inhibiting suppressive microenvironment features, or actively, by stimulating anti-tumor immune responses. To date, therapies that block inhibitory immunological signaling pathways (immune checkpoints) promoted within tumor microenvironments have demonstrated the greatest clinical benefit. The posterchild for this success has been the use of monoclonal-antibody-based therapies targeting the PD1 receptor upregulated on activated T cells, or its ligands (programmed death ligands 1 and 2 (PD-L1 and PD-L2)), commonly upregulated by tumor and tumor-associated immune cells. By limiting this interaction, anti-PD1/PD-L1 therapy can release T cells (primarily CD8^+^ T cells) from (or prevent the induction of) a state of functional exhaustion in which effector functions are significantly diminished [1].

### Acquired resistance to anti-PD1/PD-L1 immunotherapy

Although anti-PD1/PD-L1 therapy is, to date, the most effective single-agent therapy used in the treatment of cancers such as melanoma, it has been shown that as many as 60 % of patients who receive it display primary resistance [2]. More worryingly still, a recent study showed that approximately 25 % of melanoma patients who demonstrated an objective response to anti-PD1 therapy developed acquired resistance, as characterized by disease progression at a median follow-up of 21 months [3]. Unfortunately, few effective therapeutic options are available for such patients, as very little is known regarding the mechanisms by which acquired resistance to anti-PD1/PD-L1 therapy occurs [4]. In a recent edition of *The New England Journal of Medicine*, some light was shed on this by Zaretsky and colleagues, who sought to identify mutations associated with resistance to anti-PD1 therapy [5]. Here, we discuss the importance of this study, how its findings further our understanding of acquired resistance to anti-PD1, and its implications for the development of combination immunotherapeutic strategies.

## Whole-exome sequencing to determine the genetic basis of resistance mechanisms

From a cohort of 74 melanoma patients, four were selected who demonstrated an objective response to the anti-PD1 therapy pembrolizumab (also known as Keytruda; Merck), who progressed after a median of 1.8 years despite continuous therapy, and from whom tissue biopsies were available at baseline (before therapy) and after disease progression. Initial responses to anti-PD1 therapy were associated with increased CD8^+^ T-cell infiltration within tumor tissue. At relapse, however, CD8^+^ T cells were usually restricted to the tumor margin. To identify a genetic basis for the different phenotype of tumors at relapse, whole-exome, next-generation sequencing was used to compare DNA isolated from tumor cells derived from either biopsy material or early passage cell lines obtained at baseline or at the time of progression. It was revealed that two distinct pathways were affected within different progressing lesions that limited the sensitivity of tumor cells to anti-tumor immunity.

**Fig. 1:**
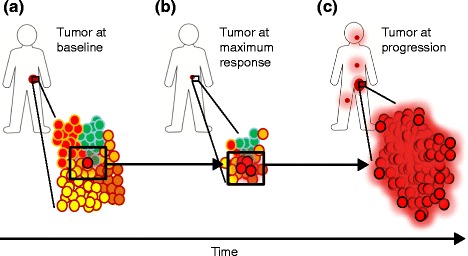
Clonal selection of occult tumor cells harboring T-cell resistance genes. **a** Tumor at baseline. *Circles* represent tumor cells and different *colors* represent intra-tumor heterogeneity with respect to genetic composition. The *red tumor cell with a black border* harbors T-cell resistance mutations. **b** Tumor at maximum response. Although the bulk of the tumor is sensitive to immunological assault as a result of anti-PD1 therapy, tumor cells harboring resistance genes are selected for, increasing the ratio of T-cell-resistant to non-resistant cells. **c** Tumor at progression. The tumor is largely composed of cells containing resistance genes. In the absence of immunological control, metastatic disease is capable of progression and metastasis

Two patients were found to harbor loss-of-function mutations within the genes encoding either Janus kinase 1 or 2 (*JAK1* or *JAK2*), key intracellular signaling intermediates necessary for sensitivity to T-cell-derived effector molecules known as interferons (IFNs). Mutations were present at relapse and were accompanied by deletion of the wild-type allele and duplication of the mutant allele, leading to a two-copy loss of wild-type *JAK*. Analyses revealed that resistant *JAK*-mutant cells were derived from tumor cells clonally selected from those present at baseline (Fig. 1). Functional characterization was carried out in vitro, revealing that cells harboring mutant *JAK* genes were capable of presenting antigen and of being recognized by cognate antigen-specific T cells. Interestingly, however, the sensitivity of the tumor cells to T-cell-derived IFNs was dramatically decreased, evidenced by reduced sensitivity to the anti-proliferative effects of IFNs, decreased signal transducer and activator of transcription 1 (STAT1) phosphorylation (an important transcription factor, phosphorylated by JAK1 and 2), and reduced upregulation of major histocompatibility complex (MHC) class I and PD-L1 in response to IFNs. The second pathway shown to promote resistance to anti-PD1 therapy was a familiar face [5]: a mutation within the gene encoding β-2-microglobulin (*B2M*), the protein product of which is necessary for the folding and transport of MHC class I molecules to the cell surface where they present antigen to T cells. This mutation was shown to prevent the recognition of tumor antigen by antigen-specific T cells.

## Primary and acquired resistance to anti-PD1 therapy in other studies

This study very effectively demonstrated that like molecularly targeted therapies, immunotherapies can select for tumor cells resistant to pathways normally vulnerable to T-cell-mediated assault in humans. This complements the findings of others who have used mouse models to show that acquired resistance to anti-PD1 therapy can develop by non-genetic means, via upregulation of additional exhaustion markers such as T-cell immunoglobulin mucin 3 (Tim3) [6]; however, it is not clear whether such effects will be observed in human disease. Other studies investigating resistance to anti-PD1 therapy have focused upon primary resistance and have suggested that a number of factors can promote T-cell resistance, such as poor tumor immunogenicity [7], defective antigen presentation and naive T-cell priming [8], limited intra-tumoral T-cell infiltration, elevated PD1 expression on tumor-infiltrating T cells representing a state of severe functional exhaustion [1], and the induction of alternative immunosuppressive pathways within the tumor microenvironment [6], such as the release of extracellular adenosine or indoleamine 2,3-dioxygenase [1]. Importantly, the majority of these findings have been made within mouse models and their relevance to human cancers has yet to be comprehensively investigated.

## Unanswered questions and future studies

The study conducted by Zaretsky et al. raises several interesting questions. First, will patients with other cancer types that respond to anti-PD1/PD-L1 develop acquired resistance with similar mutations or pathways? Melanomas tend to have an extremely high mutational burden in comparison with other tumor types, which could logically reduce the chance that *JAK* or *B2M* mutations might arise [7]. Second, are there ways of rescuing the sensitivity of patients who acquire resistance to anti-PD1 therapy via these pathways? One approach, which was investigated to a limited extent within the current study, is the use of stimulator of interferon gene (STING) agonists that are capable of promoting STAT activation independent of JAK2 [5]. Another approach might be the use of combination therapies that target tumor proliferation while also promoting immune infiltration, such as *BRAF*-targeted therapies or oncolytic viruses that have been demonstrated to replicate preferentially within cells with defective IFN signaling [9]. Third, could tumor antigen-specific CD4 T cells, or innate cells such as NK cells or myeloid cells, be targeted to promote anti-tumor immunity in tumors harboring *B2M* mutations? Answering these questions will likely provide solutions to overcome immunotherapeutic resistance.

So, is this checkmate? Is the game over for anti-PD1 therapies? Not in our opinion. Although immune checkpoint inhibitors are only entering their adolescence, they are currently proving to be without doubt the most effective therapeutic option available to promote anti-tumor immunity. Immunotherapies will become a mainstay of cancer treatment, so understanding resistance is extremely important, but we need to know more before we make our next move. Given that three out of the four patients screened within this study harbored different mutations (within two separate pathways), it seems highly likely that additional studies with larger cohort sizes might identify alternative causative pathways. The findings of such studies, when coupled with tumor-immune profiling, could be of great benefit in a predictive context to stratify patients based upon likelihood of response and to guide the rational application of combination therapies. We have seen one good move. It is now up to us to look for the next one.
